# The Effect of Personalized Shoe Insoles on Parkinson’s Disease Subjects: A Triple-Blind Randomized Controlled Trial

**DOI:** 10.3390/jcm12237204

**Published:** 2023-11-21

**Authors:** Joel Pollet, Riccardo Buraschi, Giorgia Ranica, Simone Pancera, Denise Anastasi, Rossella Fazio, Serena Monteleone, Eleonora Lena, Valeria Floridi, Franco Zucchini, Maurizio Vincenzo Falso

**Affiliations:** 1 IRCCS Fondazione Don Carlo Gnocchi, 20162 Milan, Italy; jpollet@dongnocchi.it (J.P.); rburaschi@dongnocchi.it (R.B.); spancera@dongnocchi.it (S.P.); danastasi@dongnocchi.it (D.A.); elena@dongnocchi.it (E.L.); vfloridi@dognocchi.it (V.F.); mfalso@dongnocchi.it (M.V.F.); 2 ASST Spedali Civili, 25123 Brescia, Italy; rolax@live.it; 3 ASST Papa Giovanni XXIII, 24127 Bergamo, Italy; smonteleone@asst-pg23.it; 4 Poliortopedia s.r.l., 25126 Brescia, Italy; franco@poliortopedia.it

**Keywords:** Parkinson’s Disease, rehabilitation, neurological rehabilitation

## Abstract

Subjects with Parkinson’s Disease (PD) display different motor and non-motor symptoms. Different therapies have been shown to be effective, such as plantar foot stimulation, which has proved to be effective for motor symptoms. Different stimulation methods were proposed and tested through specific devices, or insoles. Our aim was to assess the effect of a newly designed custom-made insole called PRO-STEP compared with a flat sham insole on subjects with PD. Subjects were randomized 1:1 into two arms and were asked to wear PRO-STEP or sham insoles for at least 6 h per day for 10 weeks. Participants were evaluated at four timepoints. Forty-two subjects were randomly assigned to the PRO-STEP (EG) or sham group (SG). The comparison of the EG and SG without and with insoles (T0–T1) did not show significant differences in the TUG time and in the 10MWT gait parameters. At T1, T2, and T3 TUG time, BBS, SF12-MC, and SF12-PC did not show significant differences. The satisfaction level with the PRO-STEP or sham insoles was high in both groups. PD patients were satisfied with PRO-STEP insoles; however, plantar foot stimulation is not effective from a functional perspective. Future studies should consider possible modifications to the proposed stimulation to improve its effectiveness in patients with PD.

## 1. Introduction

Idiopathic Parkinson’s Disease (PD) is a common neurodegenerative pathology mainly characterized by postural instability and gait impairments due to motor and non-motor manifestations [1,2]. All these symptoms affect patients’ quality of life, exacerbating disability and dependency levels [2]. The gold-standard treatment for this condition is represented by pharmacological therapies [3]. Concurrently, patients usually undergo neurorehabilitation sessions, aiming to maintain and possibly improve gait, balance, and posture and to acquire greater autonomy in everyday life [3,4]. Alongside traditional rehabilitation, other innovative treatments have been proposed, such as virtual reality [5], robotic rehabilitation [6], and rhythmic activities such as dance [7], Tai-Chi [8], and boxing [9]. Another treatment that is gaining importance is represented by mechanical stimulation, in particular of the foot [10]. Quattrocchi et al. showed how mechanical stimulation modifies patients’ characteristics both at the cortical level, enhancing activation and connectivity levels, and at the functional level, decreasing the Unified Parkinson’s Disease Rating Scale (UPDRS) score [11]. The proposed stimulation changes among studies and some systematic reviews analyzed the different types and effects of stimulations used [10,12]. Some studies used a passive device called a Gondola™ [13], and others used insoles with different modifications, from half-sphere elevations [14] to ribbed insole facilitation [15]. The interventions produced widespread results, from postural and balance improvements to gait modifications [10,12]. However, these studies present some limitations and unsuitable study designs, with small sample sizes and the effect of the insoles being assessed in most of the studies as an immediate effect; until now, the maximum length of these studies has been 5 weeks, but as reported by Lirani-Silva et al., a longer wearing period should be studied [14]. Previous studies present some bias in the randomization process and in the blinding of participants, personnel, and assessor that negatively affects the real effect of the interventions [12]. Thus, high-quality randomized controlled trials with more extended treatment periods are necessary to prove the effectiveness of any type of new plantar foot stimulation on patients with PD [12]. Therefore, to prove the effectiveness of a new custom-made insole called PRO-STEP, a randomized controlled trial was designed to reduce possible sources of bias. The concept of the new PRO-STEP insole exploits mechanical stimulation through the elevation of the foot’s metatarsal and digital area with a material characterized by elastic properties which imply an increased pressure over this area in the final part of the second rocker and the whole third rocker phase. The elevation of the forefoot could represent a helpful intervention to assist the lack of lower limb propulsion typical of PD [16]. Thus, this study aims to assess, through a randomized controlled trial, (1) the immediate effect, with and without PRO-STEP and conventional insoles, and (2) the effect on a more extended period on function, balance, and quality of life of the PRO-STEP insole compared to a conventional flat insole considered as a sham in patients with PD.

## 2. Materials and Methods

Our study is a two-arm, triple-blind, randomized controlled trial. The trial protocol was approved by the Fondazione Don Gnocchi Ethical Committee and conducted according to the Helsinki declaration. Study protocol was registered on clinicaltrial.gov, NCT04803565. The study lasted from March 2021 to March 2023 in the tertiary referral rehabilitation center “Spalenza-Fondazione Don Carlo Gnocchi” and was reported according to the CONSORT guidelines.

### 2.1. Selection of Patients

The patients were evaluated for eligibility by a physician specialized in Physical and Rehabilitation Medicine (PRM). Inclusion criteria were patients diagnosed with idiopathic PD, patients > 18 years, and patients able to walk independently or with a walking aid for at least 10 m. Exclusion criteria were patients with no clinical stability, other neurological diseases, pathologies, or significant alterations that may impinge gait cycle, and patients unable to sustain assessment procedures. Some patients were already on medication, and they continued the pharmacological therapy during the study period. After collecting the signed informed consent, the physician assessed patients for eligibility and administered the clinical baseline evaluation (MDS-UPDRS part 3, Hoehn and Yahr scales, and Mini-Mental examination).

### 2.2. Randomization and Blinding

A 1:1 randomization was performed through online software (random.org, accessed 15 February 2021) by a researcher not involved in the study. The subjects were allocated to the experimental group (EG) or sham group (SG). Patients were allocated according to the opaque sealed envelopes opened by the technician in charge of providing and producing custom-made and sham insoles. Patients, therapists, and outcome assessors were unaware of the type of insole assigned.

### 2.3. Outcome Assessment

The study’s primary outcome measure was the Time Up & Go (TUG) time. The TUG was implemented with an inertial measurement unit (IMU, G-Sensor, BTS Bioengineering, Garbagnate Milanese, Milan, Italy) to assess the spatiotemporal and kinematic parameters during the execution of the test. As secondary outcome measures, the 10 m walking test (10MWT) with IMU, the Berg Balance Scale (BBS), and the Short-Form 12 (SF12) were administered. Moreover, a Likert (0–4) scale was used to assess the participants’ satisfaction with the insoles. Data extracted from the IMU through the G-Studio software (ver. 3.3.22.0) during the 10MWT included speed, symmetry index, stride length, cadence, and swing phase. The physical (SF12-PC) and mental components (SF12-MC) of the SF12 were considered. There were four time-points for the outcome assessments. Two evaluations were performed before the first rehabilitation session, at T0 without insoles and at T1 with insoles. The third assessment (T2) was performed after 10 weeks. A follow-up assessment was carried out four weeks (T3) after the end of treatments. All outcome measures were evaluated at every time-point except for SF12, which was taken at T0, T2, and T3. Table 1 reports all the assessed time-points and the related evaluations.

### 2.4. Insoles

**Figure 1:**
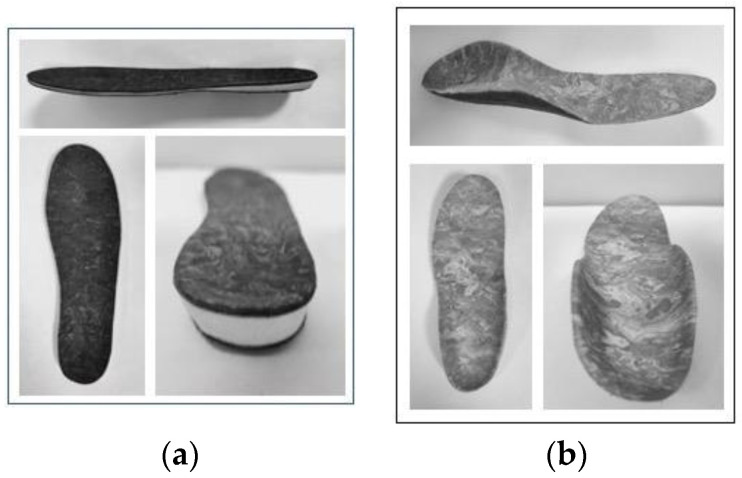
(**a**) Picture of the sham insole. (**b**) Picture of the PRO-STEP insole.

The insoles used in this study were of two types. A standard industrially manufactured insole, without customization, was used as sham (Figure 1a). The custom-made insoles (PRO-STEP, Poliortopedia, Brescia, Italy) were produced from the foot cast of the patient and then molded to fit best (Figure 1b). Both types of insoles had the same outer coating to be visually indistinguishable.

To maintain allocation blinding, a foot cast in an orthostatic position was taken from each patient after their inclusion in the study, regardless of their group. The manufacturing process of the experimental insole took about two weeks. The insole produced a mechanical stimulation on the foot through the elevation of 1 cm of the forefoot. This elevation was produced with a fiberglass foil that presents specific elastic properties. These may enhance the pressure on both feet’s digital and metatarsal areas during the final part of the second and the whole third rocker. The elastic return of the material helped the subjects during the swing phase, allowing an easier ankle dorsiflexion. The technical properties of the experimental and sham insoles are available in Supplementary Material S1.

### 2.5. Intervention

The patients in both groups were asked to wear the insoles for at least 6 h daily for 10 weeks. To verify the adherence to the prescribed hours of treatment, each patient was asked to fill in a weekly diary. The outpatient rehabilitation was delivered to both groups according to the European Guidelines for Parkinson’s Disease Rehabilitation [17,18], and the therapists’ activities were monitored through a specific form. The rehabilitative intervention lasted for 20 sessions, 2 sessions per week for 90 min (60 min of physiotherapy and 30 min of occupational therapy). At the end of the rehabilitation sessions, the patients were assigned home-based exercises of automobilization, balance, and coordination. Their activity and adherence to the treatment, including whether they wore the insole for at least 6 h, were monitored through a weekly diary collected on the last assessment. After six weeks, the follow-up assessment was performed.

### 2.6. Statistics

The study’s sample size was calculated a priori using the primary outcome, TUG, with G*Power 3.1 software. Two degrees of freedom, a power (1 − β) of 80%, an α = 5%, a minimum clinically important difference of 3.5 s [18], and a standard deviation of 3.7 s [19] were used for a two-way analysis of variance (ANOVA). The results were incremented by 20% to prevent problems due to eventual dropouts; a number of 21 subjects per group (42 subjects in total) was retrieved.

All calculations were carried out using R ver. 4.3.1 [20], and data are expressed as the mean ± standard deviation (SD) or median (first-third interquartile, 1–3 Quart) with a 95% confidence interval (95%CI). Across-group differences in demographic data and general gait parameters were detected using the independent t-tests. The immediate effect of the insole and the effect of the experimental insole at T1, T2, and T3 were calculated with the two-way ANOVA to assess differences between groups (experimental insole vs. sham-insole) with and without insoles. A Q-Q plot and the Shapiro–Wilk test were used to check the normality of the distributions, and we checked residuals to verify assumptions for the linear model and the presence of influential cases. An intention-to-treat analysis was performed and maximum likelihood estimation via the expectation-maximization algorithm was used for imputation of missing values.

## 3. Results

**Figure 2:**
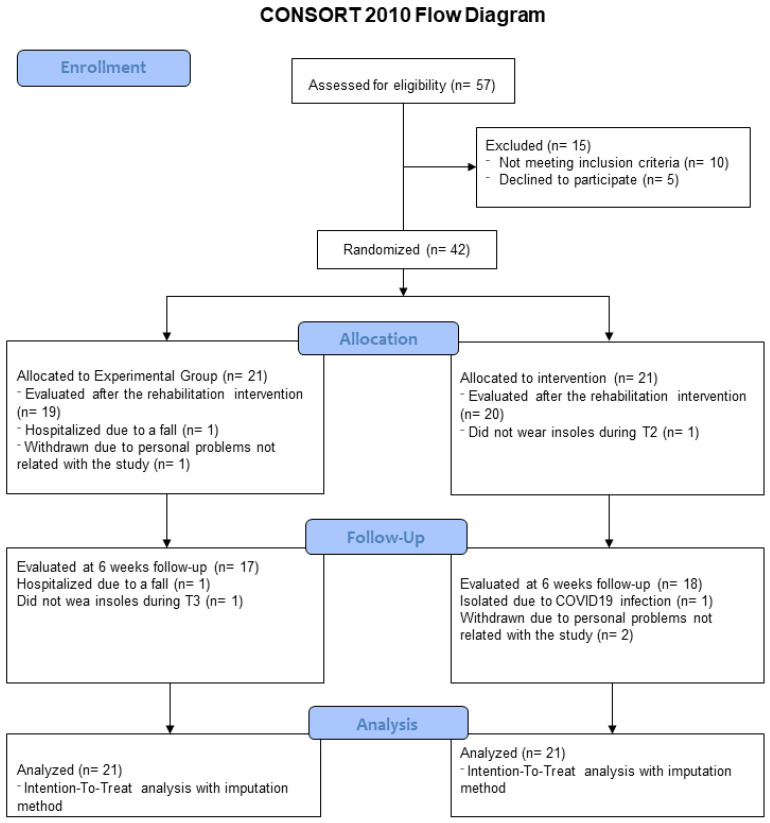
Study flow chart.

Fifty-seven subjects were evaluated for inclusion in the trial. Forty-two patients were enrolled for the study: 21 in the EG and 21 in the SG. During the study, two participants were lost in the EG group and one in the SG. The reasons for dropout are reported in the study flow chart (Figure 2). The two groups did not show any significant difference in any of the baseline parameters considered (Table 2). Participants in both groups adhered to the treatment by wearing the insoles most of the time; in particular, EG subjects wore the insoles 79% ± 17% and SG subjects wore them 83% ± 16% of the time.

### 3.1. Immediate Effect of Insoles Application

**Figure 3:**
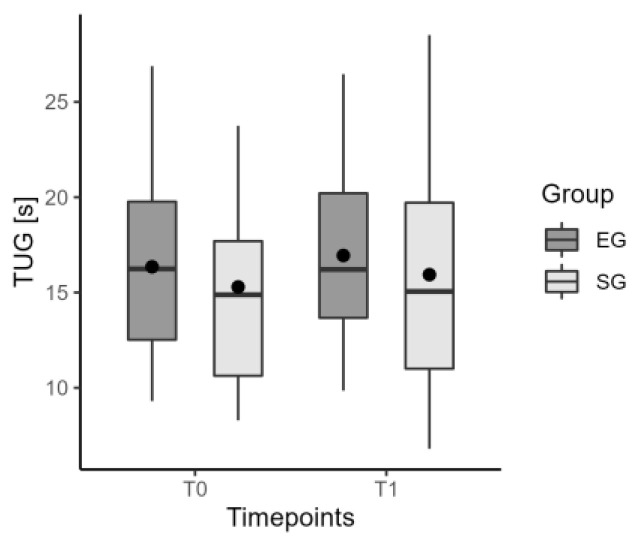
The values of median, mean, and quartiles of TUG time in EG and SG for the immediate effect.

**Figure 4:**
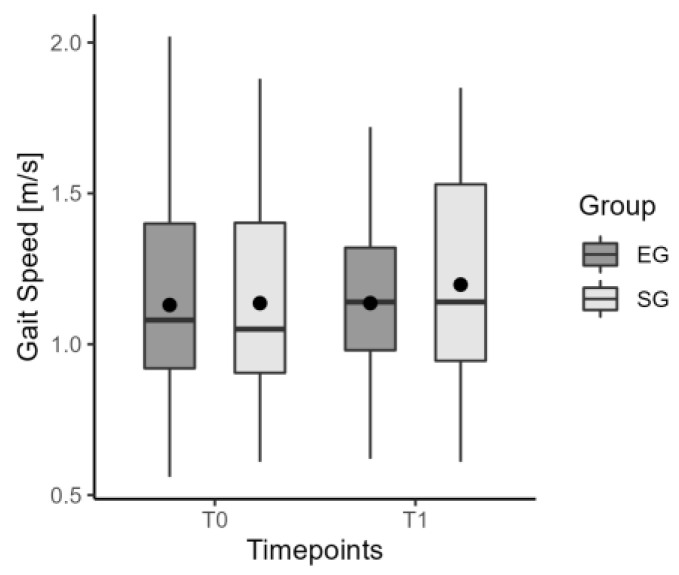
The values of median, mean, and quartiles of gait speed in EG and SG for the immediate effect.

Neither insole showed any immediate change (T0–T1) in the TUG time (Figure 3). Moreover, spatio-temporal parameters of gait such as speed (Figure 4), step length, swing phase, cadence, and symmetry index, did not show any difference with and without insoles. Table 3 summarizes data on the immediate effect for the considered parameters.

### 3.2. Long-Term Effect of the Insoles

**Figure 5:**
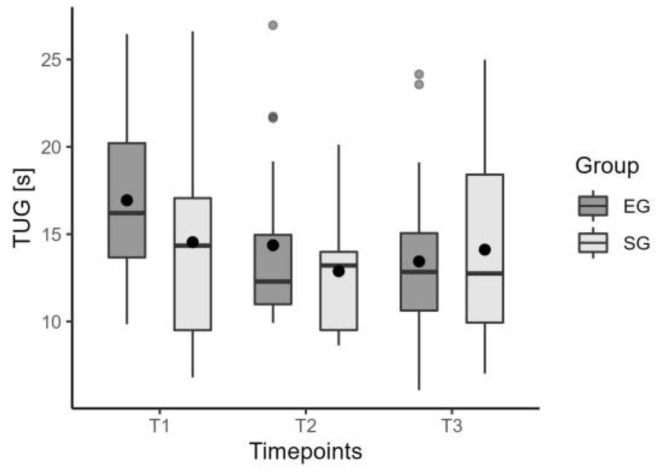
The values of median, mean, and quartiles of TUG time in EG and SG for the long-term effect.

**Figure 6:**
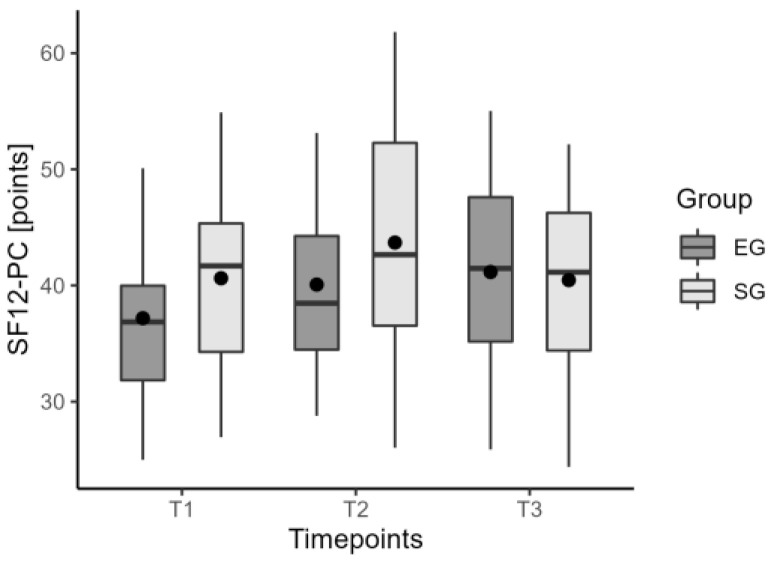
The values of median, mean, and quartiles of Short Form-12 Physical component in EG and SG for the long-term effect.

The PRO-STEP insoles did not show significant improvement compared to the sham insoles after 6 weeks (T2) and at the one-month follow-up (T3). TUG time did not differ significantly, *p* = 0.61 (Figure 5). The other functional outcomes considered, BBS, 10MWT parameters, SF12-PC (Figure 6), and SF12-MC, did not produce significant results. Moreover, spatio-temporal parameters did not produce significant results. Table 3 shows data on the long-term effects.

### 3.3. Satisfaction with Insoles

**Figure 7:**
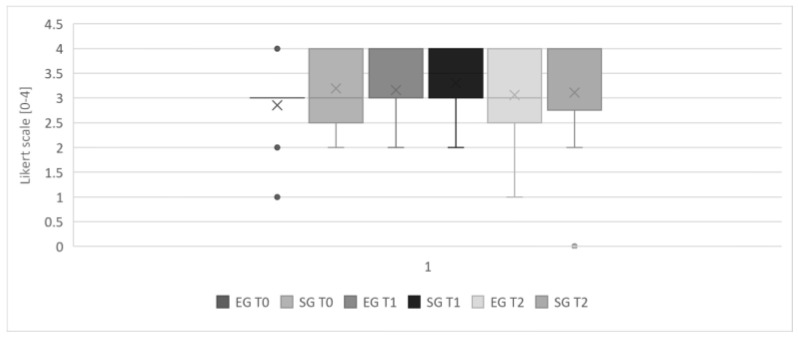
Satisfaction level of the two groups during the different time-points of the study (× = mean value).

The satisfaction level for both insoles was considerably high and remained high for the whole duration of the study. The two groups did not significantly differ in the satisfaction level (Figure 7).

## 4. Discussion

In this study, we evaluated how customized insoles could improve the function, gait, and balance of patients with PD. The results showed no significant improvement using the custom-made insole compared to a sham conventional insole.

Patients with PD well accepted the proposed intervention; almost everyone had a high level of satisfaction on the Likert scale. The scientific protocol did not include a cut-off for treatment adherence, but the analysis of the subject’s diaries showed a high adherence to the treatment, with an average adherence of 81% in both groups.

This study shows no differences in the immediate use of insoles in the parameters considered. Previous studies showed significant changes only in a limited number of parameters [19]. On the contrary, other authors showed significant changes in different parameters [15]. However, considering a longer observation period, our study reports a non-significant change in any gait parameter. In contrast, a previous study reported a significant change after 5 weeks of stimulation only in the stride length [14]. Nonetheless, in our study, the proposed stimulation may not have been sufficient to produce a significant change in the participants’ performance. In fact, the interventions proposed by other authors presented a strong haptic stimulation in specific anatomical landmarks [19,21], while the insole tested in this study did not present such a stimulation, but a whole stimulation of the metatarsal and digital area. Therefore, increased stimulation or the inclusion of a specific haptic stimulation should be tested to verify a possible combination with the stimulation proposed in this study.

Previous studies in this field assessed the effect of different kinds of insoles with a wide variety of outcome measures, without a focus on significant outcome measures proposed by previous studies or systematic reviews. Instead, we decided to focus on those outcome measures identified as significant in systematic reviews [10,12] and trials [14,19] on similar interventions. With previous studies in mind, we also included an evaluation of functional [22] and quality of life [23] measures that were not considered by other authors investigating these kinds of interventions.

To the best of our knowledge, this is the first study presenting a randomized controlled design with blinding and an a priori sample size calculation. We decided to base our sample on a functional test due to the central role of functional performance in the measure of interventions on PD [4]. With regards to the choice of a functional outcome, it can be argued that gait speed would be a better choice, and thus that the effect of the PRO-STEP should be focused on gait. However, from a rehabilitative point of view, an increased gait speed should also increase function to be effective in patients’ daily life activities.

This study presents some limitations compared to previous studies. First, compared to Lirani-Silva et al. [14], our study did not provide an evaluation of the subjects’ plantar sensation. This evaluation should be included in future developments with a new insole design. Second, other studies provided an evaluation of subjects without insoles at all time-points. In contrast, we did not provide such an evaluation, as our custom-made insole was intended to be permanent in subjects’ shoes.

All patients were in the early PD stage (H&Y stage 2), and their gait speed was 1.13 m/s (experimental group) and 1.14 m/s (sham group). According to these features, patients had no extreme difficulties in gait and balance (i.e., the EG and SG groups’ mean Berg Balance Scale scores were 44.90 and 47.00, respectively; for values above 45, patients should be able to move safely without risk of fall), so seeing improvements may have been more challenging. Moreover, all patients were exposed to physiotherapy, making the assessment of the real effect of the insoles without rehabilitation impossible. Finally, there was not monitoring of patients’ medication changes during the RCT, with the population only being evaluated during the “ON” phase.

Further studies should include a population with a higher level of disability to verify the possible effect of PRO-STEP on patients with a large level of impairment. Moreover, a waiting list group wearing PRO-STEP without being exposed to physiotherapy and drug monitoring is required for future research.

## 5. Conclusions

This randomized controlled trial compared the effect of a custom-made insole to a sham insole on functional, gait, and quality of life parameters in patients with PD. Despite the well tolerated intervention, the custom-made insoles did not provide further improvements in the outcome considered at any of the assessed time-points. In future studies, the design of the proposed custom-made insoles should be modified in order to produce significant effects in patients with PD.

## Figures and Tables

**Table 1:** Assessment times and outcome measures performed.

	Assessment Time-Points			
	T0	T1	T2	T3
Assessment time	Before the first rehabilitation session without wearing PRO-STEP (week: 0)	Before the first rehabilitation session wearing PRO-STEP (week: 0)	After the last rehabilitation session wearing PRO-STEP (week: 10)	One month after the last rehabilitation session wearing PRO-STEP (week: 14)
Outcome measures	(1) TUG (with IMU) (2) 10MWT (with IMU) (3) BBS (4) SF12-PC (5) SF12-MC	(1) TUG (with IMU) (2) 10MWT (with IMU) (3) BBS	(1) TUG (with IMU) (2) 10MWT (with IMU) (3) BBS (4) SF12-PC (5) SF12-MC	(1) TUG (with IMU) (2) 10MWT (with IMU) (3) BBS (4) SF12-PC (5) SF12-MC

**Table 2:** Mean and standard deviation of demographic parameters of the two groups.

Parameter	Experimental Group (N = 21)	Sham Group (N = 21)	*p* Value
Sex	F 6 (28.6%) M 15 (71.4%)	F 7 (33.3%) M 14 (66.7%)	1
Age (years)	72.0 (7.05)	72.0 (5.02)	0.659
Height (cm)	169 (7.90)	166 (9.07)	0.181
H&Y (points)	2.05 (0.72)	1.95 (0.97)	0.635
UPDRS III (points)	27.1 (12.6)	28.0 (13.6)	0.93
BBS (points)	44.9 (9.05)	47.00 (7.32)	0.659
Speed (m/s)	1.13 (0.35)	1.14 (0.37)	0.801
TUG (s)	16.35 (5.19)	15.29 (5.35)	0.654

TUG: Timed Up and Go test, BBS: Berg Balance Scale, H&Y: Hoen and Yahr scale.

**Table 3:** Mean (standard deviation) of clinical and instrumented data of the experimental and sham groups, at baseline (T0), immediately after insoles delivery (T1), 10 weeks after the first evaluation (T2), and 14 weeks after the first assessment (T3); values of F and the *p* value are reported for the two-way ANOVA performed between groups and assessment time; no post-hoc analysis was performed due to the lack of significance of the results.

	T0		T1		T0 vs. T1		T2		T3		T1 vs. T2 vs. T3	
					F (Group × Time)	*p* Value (Group × Time)					F (Group × Time)	*p* Value (Group × Time)
	EG	SG	EG	SG			EG	SG	EG	SG		
TUG	16.35 (5.19)	15.29 (5.35)	16.94 (4.61)	15.94 (6.80)	0.047	0.829	14.37 (4.71)	12.88 (3.23)	13.44 (4.91)	14.11 (5.40)	1.162	0.317
BBS	44.90 (9.05)	47.00 (7.32)	-	-	-	-	47.18 (9.07)	51.12 (6.25)	48.93 (9.72)	48.76 (7.12)	0.043	0.958
SF12-PC	37.18 (6.72)	40.62 (8.32)	-	-	-	-	39.99 (7.48)	46.51 (9.11)	42.35 (8.50)	40.46 (8.28)	0.756	0.472
SF12-MC	43.09 (11.81)	43.09 (11.07)	-	-	-	-	47.35 (9.93)	45.57 (12.23)	44.93 (9.31)	44.17 (11.69)	0.029	0.971
Instrumented indexes												
Speed (m/s)	1.13 (0.35)	1.14 (0.37)	1.14 (0.30)	1.25 (0.36)	0.127	0.722	1.23 (0.26)	1.30 (0.29)	1.21 (0.28)	1.18 (0.31)	0.056	0.946
Cadence (step/min)	109.08 (15.89)	105.73 (15.06)	110.59 (14.36)	110.86 (13.91)	0.313	0.755	110.12 (13.03)	107.99 (10.56)	111.94 (13.83)	109.37 (11.13)	0.030	0.970
Stride Length (m)	1.26 (0.31)	1.29 (0.30)	1.25 (0.27)	1.36 (0.30)	0.067	0.797	1.35 (0.22)	1.45 (0.26)	1.32 (0.22)	1.31 (0.31)	0.030	0.970
Left Swing Phase (% cycle)	39.34 (4.22)	37.95 (3.24)	38.99 (4.05)	37.77 (3.39)	0.017	0.898	38.50 (3.44)	36.66 (2.70)	38.36 (4.24)	39.04 (2.53)	1.106	0.335
Right Swing Phase (% cycle)	39.37 (3.32)	38.84 (2.47)	40.05 (4.30)	39.01 (3.32)	0.219	0.641	40.29 (3.12)	38.40 (3.28)	40.18 (3.89)	38.38 (2.79)	0.060	0.9421
Symmetry Index	88.53 (7.42)	89.88 (9.87)	89.14 (8.32)	87.83 (7.71)	0.477	0.492	91.64 (5.78)	90.87 (7.01)	86.15 (10.08)	89.78 (9.58)	0.9158	0.4036

EG: experimental group, SG: sham group, TUG: Timed Up and Go test, BBS: Berg Balance Scale, SF12-PC: Short-Form 12 Physical Component, SF12-MC: Short-Form 12 Mental Component.

## Data Availability

The data presented in this study are available upon request from the corresponding author.

## Supplementary Materials

The following supporting information can be downloaded at: https://www.mdpi.com/article/10.3390/jcm12237204/s1, Table S1: Resume of the structural, manufacturing, and functional characteristics of the pro-ergonomic foot insole (own elaboration). Table S2: Resume of the structural, manufacturing and functional characteristics of the pro-ergonomic foot insole (own elaboration).
