# Supplementary material for: The Effect of Personalized Shoe Insoles on Parkinson’s Disease Subjects: A Triple-Blind Randomized Controlled Trial
Source: J Clin Med. 2023 Nov 21;12(23):7204. doi: 10.3390/jcm12237204 (PMC10707212; doi:10.3390/jcm12237204)
Supplement: Supplementary file 1 [file jcm-12-07204-s001.zip › jcm-2706765-supplementary.pdf]

Supplementary Material S1

The insoles used in this study were of 2 types. A traditional ergonomic insole (Figure 1a), built on polyurethane mould with structural and functional characteristics summarized in Table SI.

Table S1. Resume of the structural, manufacturing, and functional characteristics of the pro-ergonomic foot insole (own elaboration).

**Structural and manufacturing characteristics**	**Functional characteristics**
• Base: 1,15 mm thick imitation leather.<br>• Structure: fiberglass/resin composite [thickness 1,2 mm].<br>• Temperature of work= 90/95° C, [density= 900 Kg/m*, biaxial].<br>• Coating: thermoformable Eva [thickness 2 mm, shore 35, [Temperature of work= 110/120°].	• Pro-ergonomic insole with physiological support of the medial plantar arch.<br>• Thermoformable shell consisting of a reinforced base in fiberglass and resin, covering the entire surface of the foot.<br>• Lacking elastic properties on the forefoot (absence of payback during push-off and initial swing).

The custom-made insole with pro-dynamic properties PRO-STEP (Poliortopedia, Brescia, Italy) (see Figure 1b) was created on a preliminary polyurethane cast of the patient's foot and with structural and functional characteristics summarized in Table S2. The functional profile of the experimental Pro-Step has specific dynamic properties, conditioned by two intrinsic characteristics of the insole: i) the thickness of the fiberglass constituting the shell that affects the degree of rigidity and resilience of the insole, called K factor; ii) the amount of elevation forefoot (metatarsal and digital area), respect to the midfoot and rearfoot, called B factor. This elevation should modulate the activation of foot intrinsic muscles. The elastic properties and the intrinsic muscle activation can help gait progress in the 2nd-3rd rocker.

Table S2. Resume of the structural, manufacturing and functional characteristics of the pro-ergonomic foot insole (own elaboration).

**Structural and manufacturing characteristics**	**Functional characteristics**
• Base: 1,15 mm thick imitation leather.<br>• Structure: composite of glass fiber and multi-layer epoxy resin according to the K factor [thickness 1,2 mm, density 750 g/m*, triaxial].<br>• Coating: thermoformable Eva [thickness 2 mm, shore 35, Twork= 110/120°].	**K factor**<br>• Outline the thickness of the fiberglass constituting the shell of the footinsole (number of fiberglass sheets).<br>• Factor conditioning the degree of rigidity or resilience of the insole.<br>• Affects the soft or hard stop in the rolling phase of the gait in the 3rd-4th rocker phase.<br>• Variable chosen according to the degree of dynamic balance sought in the alternating plantigrade stance.<br>**B factor**<br>• Distance between the tip of the insole (digital area) and the surface support.<br>• Factor conditioning the inertial push forward in the 3-4th rocker phase.<br>• B1 = 1cm, B2 = 3cm, B3 = 5cm.

To prevent discrimination between sham and experimental insoles and preserve the blind allocation of the sample, the polyurethane insole cast was acquired on all patients recruited at the end of their inclusion in the study. Starting from the acquisition of the plantar cast, the manufacturing of the insole lasted about 2 weeks. Due to its resilience characteristics (K1 used in this study) and its design determined by the B factor (B2 used in this study), the PRO-STEP insole is able to stimulate the activation of the tonic muscles of the foot naturally and in compliance with the biomechanics of the gait (triceps surae and posterior tibialis) in the 2nd-3rd rocker phase of the gait, favoring through its elastic return an easier dorsiflexion of the ankle in preswing, and initial swing phases.
